# Approach to the Patient With Treatment-resistant Acromegaly

**DOI:** 10.1210/clinem/dgac037

**Published:** 2022-01-28

**Authors:** Eva C Coopmans, Aart J van der Lely, Sebastian J C M M Neggers

**Affiliations:** Department of Medicine, Section Endocrinology, Pituitary Center Rotterdam, Erasmus University Medical Center, 3000 CA Rotterdam, the Netherlands; Department of Medicine, Section Endocrinology, Pituitary Center Rotterdam, Erasmus University Medical Center, 3000 CA Rotterdam, the Netherlands; Department of Medicine, Section Endocrinology, Pituitary Center Rotterdam, Erasmus University Medical Center, 3000 CA Rotterdam, the Netherlands

**Keywords:** acromegaly, pituitary, medical treatment, somatostatin analogs, pasireotide, pegvisomant, clinical case, surgery and radiotherapy

## Abstract

Although most tumors in patients with acromegaly are benign and are cured or controlled by surgery and/or first-generation somatostatin receptor ligands therapy, some can behave more aggressively and are resistant to these standard therapies. Acromegaly, if left untreated, is a rare and chronic disorder, commonly caused by a GH-producing pituitary adenoma and is associated with significant comorbidities and an increased mortality. Transsphenoidal surgery is considered the mainstay of acromegaly management, but medical therapy has an increasingly important role. However, disease activity is not fully controlled in a significant number of patients treated with surgery and/or high-dose first-generation somatostatin receptor ligand monotherapy. In these circumstances, therefore, repeated surgery, second-line medical therapy, and radiotherapy, alone or combined as multimodal therapeutic strategies should be considered, in a patient-centered perspective.

## Case

**Figure 1.**
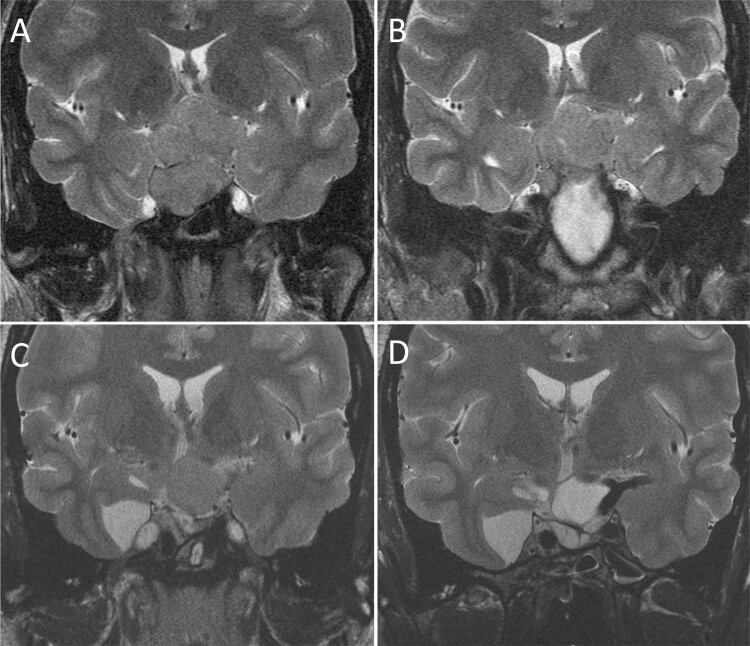
T2-weighted MRI scans at diagnosis, after first surgery, and before and after initiation of pasireotide LAR. T2-weighted MRI scan shows at diagnosis a large sellar mass with impingement of the optic chiasm and floor of the third ventricle and invasion into cavernous sinuses, right more than left (A). Postoperatively, T2-weighted MRI scan shows a large suprasellar tumor remnant (B). T2-weighted MRI scans showing tumor volume reduction from 8086 mm^3^ (C) before initiation of pasireotide to 5896 mm^3^ (D) during 15 months of treatment and increased T2-signal intensity after initiation of pasireotide LAR treatment: ROI adenoma/normal pituitary ratio before pasireotide LAR treatment was 1.2 (C) and increased to 2.0 (D). Abbreviations: LAR, long-acting release; MRI, magnetic resonance imaging; ROI, region of interest.

A 28-year-old man with acromegaly was referred to our pituitary center after transsphenoidal surgery, postoperative medical treatment with high-dose first-generation somatostatin receptor ligand monotherapy (SRL, lanreotide autogel [ATG]), and a second surgical procedure (via transcranial approach). He presented initially to a local community hospital at the age of 27 because of changes over the past 5 years in his physique, such as coarsening of facial features and acral enlargement. He also complained of fatigue, excessive sweating, and erectile dysfunction. His medical history and family history were otherwise unremarkable. His preoperative laboratory testing revealed an elevated IGF-1 2.2 × upper limit of normal (ULN), a glucose-suppressed nadir GH level of 5.9 µg/L, prolactin 40.0 µg/L (normal > 36.0 µg/L), TSH 1.2 mU/L (normal 0.4-4.3 mE/L), fT4 7.3 pmol/L (normal 13.5–24.3 pmol/L), LH 1.0 U/L (normal 1.0-5.5 E/L), FSH 0.3 U/L (normal 0.8-5.1 E/L), testosterone 3.3 nmol/L (normal 10.0-30.0 nmol/L), and morning cortisol 85 nmol/L (normal, >250 nmol/L). The 11-deoxy cortisol level after metyrapone was low (110 nmol/L, normal > 200 nmol/L). Magnetic resonance imaging (MRI) of the pituitary gland revealed a large sellar mass with impingement of the optic chiasm and floor of the third ventricle and invasion into cavernous sinuses, right more than left (Fig. 1A). Visual field examination revealed bitemporal hemianopsia. He underwent endonasal transsphenoidal tumor mass reduction. Histology of the tumor specimen confirmed a somatotroph tumor and somatostatin (SST) receptor subtyping using an immunoreactivity score (IRS) of 17 to 18, membranous expression of IRS 1 for SST_2_ receptor and IRS of 12 for SST_5_ receptor. Postoperatively, visual field defects were restored, although IGF-1 levels remained elevated and there was a large suprasellar tumor remnant (Fig. 1B). He recommenced his lanreotide ATG 120.0 mg every 4 weeks’ treatment for at least 6 months. Preoperatively hormone replacement therapy for secondary adrenal insufficiency, hypothyroidism, and hypogonadism was initiated. After 7 months, lanreotide failed to decrease IGF-1 concentrations by > 20% or induce tumor shrinkage (tumor volume change of < 25%) and he underwent a second surgical procedure via the transcranial route. Postoperatively, unfortunately, a significant tumor remnant was still present, encasing the optic tract and basal and internal carotid arteries. Also, the IGF-1 levels remained elevated during the high-dose lanreotide ATG therapy, necessitating additional therapy. We postulate that after gross total tumor resection or debulking in this patient, this may have increased the likelihood of achieving biochemical disease control with first-generation SRLs. This hypothesis is built upon a retrospective study that shows that gross total tumor resection or debulking increases the likelihood of achieving biochemical control with first-generation SRLs in patients with adenomas that were not amenable to complete surgical resection and in whom primary first-generation SRL therapy was unable to achieve good biochemical control (1).

## Background

Acromegaly is a rare chronic endocrine disorder characterized by hypersecretion of GH and IGF-1, most often as a result of a GH-producing pituitary adenoma (2). If untreated, acromegaly leads to systemic manifestations that are associated with significant comorbidities and an increased mortality, such as cardiovascular diseases, hypertension, glucose tolerance or type 2 diabetes, hypopituitarism, and possibly more malignant neoplasms (3).

Transsphenoidal surgery is the recommended first-line treatment because it represents the optimal opportunity for cure with a rapid reduction of GH concentrations and relatively low complication rates (4, 5). In the consensus statement on therapeutic outcomes, it is recommended that medical therapy in acromegaly patients is advised for patients with persistent disease activity despite surgical resection of the adenoma as well as patients that are unfit or decline surgery (4). For patients with persistent disease after surgery, both first-generation SRLs octreotide long-acting release (LAR) and lanreotide ATG represent first-line medical therapy in acromegaly (4). Despite significant medical and surgical advances, cure or long-term biochemical control is achieved in fewer than 65% of patients who undergo surgery (6, 7), and only approximately 45% of patients treated with first-generation SRLs reach biochemical control (8).

Most tumors in patients with acromegaly are benign and can be controlled by surgery and/or first-generation SRL therapy (9), although some are resistant to these standard therapies. Biochemical response to treatment with first-generation SRLs was considered as (1) biochemical response, defined as a normalized IGF-1 (IGF-1 ≤ 1.3 × ULN) after at least 6 months of treatment; (2) partial resistance, defined as a > 20% relative reduction of IGF-1 without normalization; and (3) nonresponse (ie, resistance), defined as a failure to decrease IGF-1 concentration by > 20%, which represents the intra-assay variability. Biochemical response is independent of normalized GH (GH < 1.0 µg/L) concentration. For evaluation of tumor response to first-generation SRL treatment, tumor volume change of ≥ 25% after at least 3 months of treatment was considered significant. Management of patients with treatment resistant acromegaly is complex and costly and requires a comprehensive multidisciplinary approach to provide the best standard of care, which should be always individualized, according to the patient’s needs.

### Approach to Patient With Treatment-resistant Acromegaly

In patients with acromegaly all treatment modalities try to achieve: (1) biochemical control; (2) amelioration of signs and symptoms; (3) reversal of the comorbidities and mortality risk; and (4) control of local tumor effects. Management of a patient with treatment-resistant acromegaly is challenging, requiring a comprehensive multidisciplinary approach considering patient characteristics (IGF-1 and GH levels, tumor size and invasiveness, symptoms and comorbidities, patient preference, and the cost-benefit ratio of the treatment). The therapeutic regimens are usually multimodal and include surgery and medical therapy, and even stereotactic radiotherapy could be suggested in some cases to achieve previously outlined goals.

### Repeated Surgery

In modern practice, majority of patients undergo transsphenoidal surgery, whereas transcranial approaches are only required in a few patients with tumor masses predominantly outside the sella turcica (10). Neurosurgery has a significantly better outcome in microadenomas and intrasellar macroadenomas compared with macroadenomas (Ø >1 cm). In centers with experienced neurosurgeons, biochemical remission rates of 80% in microadenomas and intrasellar macroadenomas can be achieved (11). The need for surgical reexploration depends on the size and location of the tumor remnant. In general, reoperation is usually reserved for tumor remnants that can be completely resected, for debulking to enhance efficacy of adjuvant therapies, or when impingement of the optic chiasm is still present (12). Besides, surgical reexploration is recommended for patients with severe side effects of or intolerance to adjuvant medical therapies.

### Medical Therapy

Medical therapy is recommended as adjuvant therapy for all patients with persistent disease despite surgery. Finally, medical therapy should be considered as a first-line treatment in those patients with a low chance of surgical cure or are unfit or decline surgery.

**Figure 2.**
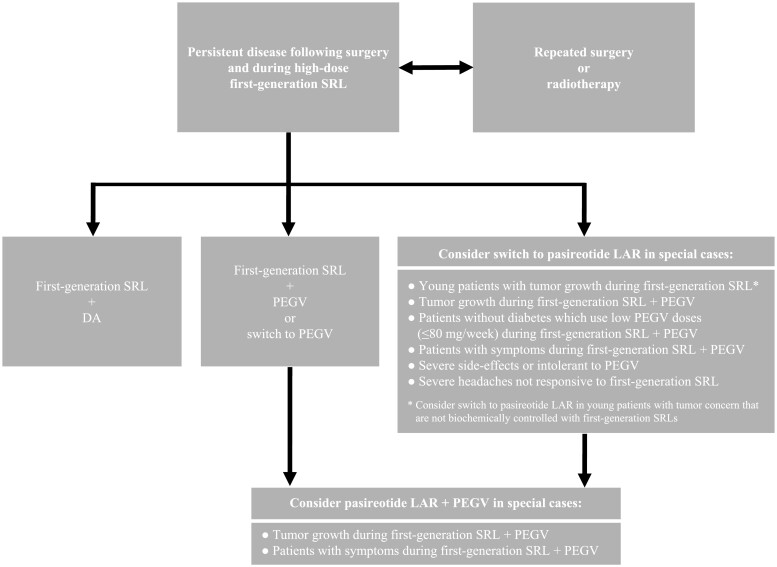
Proposed algorithm for the management of acromegaly patients with persistent disease following surgery and first-generation SRL. Repeated surgery is usually reserved for tumor remnants that can be completely resected, for debulking to enhance efficacy of adjuvant therapies, or when impingement of the optic chiasm is still present. Besides, surgical reexploration is recommended for patients with severe side effects of or intolerance to adjuvant medical therapies. Radiation therapy should be considered in patients with biochemically persistent disease and/or tumor growth despite (repeated) surgery or medical therapy. Abbreviations: DA, dopamine agonist; LAR, long-acting release; PEGV, pegvisomant; SRL, somatostatin receptor ligand.

In patients with acromegaly inadequately controlled after surgery and first-line medical therapy, second-line medical treatment options are dopamine agonists (eg, cabergoline), GH-receptor antagonists (pegvisomant [PEGV]), and the second-generation SRL pasireotide LAR. In Fig. 2, we provide recommendations for the management of acromegaly in patients with persistent disease following surgery and first-generation SRL.

#### Dopamine agonist

The expression of dopamine receptor subtype 2 by GH-producing pituitary adenomas represents the rationale for the use of dopamine agonists, independent of the presence of hyperprolactinemia (13). Currently, 2 different drugs are available: bromocriptine and cabergoline. Cabergoline is currently the most used because of better patient compliance (weekly vs daily administration of bromocriptine), and better tolerated because of reduced side effects (eg, dizziness, gastrointestinal discomfort, hypotension). Because of the modest efficacy of dopamine agonists in acromegaly, cabergoline monotherapy can be considered as first-line medical therapy only for those with modestly elevated GH and IGF-1 levels (IGF-1 < 2.5 × ULN) (4, 13, 14). The consensus statements (4) recommend the addition of cabergoline to continued first-generation SRLs treatment as second-line medical therapy for patients with inadequate control on first-generation SRL therapy (if IGF-1 < 2.5 × ULN). In contrast to the consensus statements, we recommend combination therapy with cabergoline only if IGF-1 levels are moderately elevated (IGF-1 ≤ 1.5 × ULN) because IGF-1 normalization has been seen mainly in these patients (13). Cabergoline was shown to induce potential tumor shrinkage, in particular in those acromegaly patients with GH- and prolactin-cosecreting adenomas. Data on a combination treatment of cabergoline with PEGV are limited, but this may be an option in patients who experience side effects or are intolerant to first-generation SRLs. Despite a potential benefit of dopamine agonist therapy in addition to first-generation SRLs or PEGV (15), no data from prospective studies on tumor growth in unselected or naive acromegaly patients are available to date.

#### Pegvisomant

PEGV is a pegylated form of a modified human GH analog that competitively blocks GH receptors, thus preventing binding of endogenous GH and resulting in a dose-dependent reduction of IGF-1 production. In contrast to SRLs and dopamine agonists, PEGV does not reduce GH secretion by the pituitary tumor but effectively blocks the effects of GH at the tissue level. Efficacy of PEGV monotherapy in normalizing IGF-1 levels is reported to be up to 95% of patients (16, 17), provided that appropriate dose titration of PEGV is applied. However, PEGV monotherapy does not reduce tumor size. Patients who need high doses of PEGV (dose > 30.0 mg/d, exceeding the highest allowed dose of 30.0 mg daily that is mentioned on the label) to normalize IGF-1 have more aggressive disease, as they are younger, have higher baseline IGF-I levels, and had a higher incidence of hypertension, sleep apnea, diabetes, and overweight (18). Treatment with high doses of PEGV are only used in experienced pituitary centers and is not part of a consensus on treatment of acromegaly (4). In a global noninterventional safety surveillance study of long-term treatment outcomes in patients treated with PEGV (ACROSTUDY), there were no unexpected safety issues in patients treated with high doses of PEGV, and the incidence and the type of adverse events were not different between patients need higher (mean 44 ± 12.5 mg/d) or lower (mean 7.5 ± 2.5 mg/d) PEGV dosing (18). PEGV treatment does improve glucose metabolism in patients with acromegaly by reducing insulin resistance (19, 20). There is a small risk of significant liver enzyme elevations and close monitoring of liver function is recommended, although liver failure has not been reported (21). PEGV monotherapy should be considered as a second-line treatment of choice for patients without biochemical response to monotherapy with first-generation SRLs for whom glycemic control is challenging (4). For patients who have no access to PEGV or if the patient’s health insurance is unable to cover the costs of (high-dose) PEGV treatment (average annual cost per patient is €60 000), we would recommend combination treatment with first-generation SRL and cabergoline.

The consensus statements (4) recommend a combination of first-generation SRL and PEGV in patients who remain uncontrolled with first-generation SRL and with impaired glucose tolerance and tumor concern. In contrast to the consensus statements, we recommend first-generation SRL and PEGV combination therapy as the second-line treatment of choice in nonresponders to first-generation SRL because it can lead to IGF-1 normalization in most patients (22, 23). Although PEGV monotherapy does not reduce tumor size, combination therapy has the potential advantage that it may result in tumor size control or even tumor shrinkage in most patients (22, 24). Another advantage of combination therapy is that it reduces the PEGV dosage needed to normalize IGF-1 levels by approximately 50% compared with PEGV monotherapy, leading to a reduction in injection frequency or daily dose (22, 25, 26). In a single-center prospective study in 52 first-generation SRL controlled and uncontrolled patients (27), a combination of low-dose octreotide LAR (10.0 mg) or lanreotide ATG (60.0 mg) every 4 weeks combined with weekly PEGV (40.0-160.0 mg/wk) achieved a biochemical control rate of 96%. Hence, combination therapy may increase adherence and reduce patients’ signs and symptoms at a considerably lower cost compared with combination regimens of higher dose SRL and weekly PEGV or low-dose SRL and daily PEGV. In general, if patients who are already biochemically controlled with first-generation SRL monotherapy do not report improvements in clinical symptoms during this therapy, we recommend initiating first-generation SRLs and PEGV combination therapy (28), except in patients who show poor control of diabetes during monotherapy with first-generation SRLs.

#### Pasireotide LAR

Pasireotide LAR is a long-acting somatostatin multireceptor ligand with a unique receptor binding profile. Compared with first-generation SRLs, which show the highest affinity to SST receptor subtype 2 (SST_2_), pasireotide binds with higher affinity to SST_5_ receptor, followed by 2, 3, and 1. Efficacy of pasireotide LAR monotherapy in normalizing IGF-1 is higher than that of octreotide LAR in medically naïve patients (29). The effects of pasireotide LAR and octreotide LAR therapy on GH levels reduction were, however, superimposable (29). Among patients inadequately controlled with octreotide LAR, biochemical control rates are up to 20% higher with pasireotide LAR, even when using the current cutoffs for biochemical control (30, 31). In patients previously treated with first-generation SRLs, tumor volume reduction occurred more often in those on pasireotide LAR 40 mg (19%) and 60 mg (11%) than in those inadequately controlled on high-dose, first-generation SRLs (2%) (31). However, in medically naïve patients, pasireotide LAR and octreotide LAR therapy had a similar effect on tumor volume reduction (29). During the extension phase of the latter study, a greater proportion of patients receiving pasireotide LAR achieved clinically significant (≥ 20%) tumor volume reduction after 6 months of treatment (54% vs 42% patients receiving octreotide LAR) (32). The consensus statements (4) advocated the position of pasireotide LAR monotherapy as second-line therapy: only if patients are not controlled on first-generation SRLs and if there is a clinically relevant residual tumor that is unsuitable for resection, patients should be switched to pasireotide LAR therapy. This recommendation is in line with the European Medical Association-approved label of pasireotide LAR in which it is considered a second-line option, whereas according to the US Food and Drug Administration-approved label of pasireotide LAR, its use is also allowed as first-line medical treatment of acromegaly. In addition to the consensus statements (4), we recommend pasireotide LAR monotherapy as second-line treatment in patients who show tumor growth during PEGV, alone or in combination with first-generation SRLs, or experience symptoms of active acromegaly during first-generation SRL and PEGV combination therapy. In those patients with tumor growth, pasireotide can be considered as a treatment step before starting with radiotherapy. In patients who experience side effects or are intolerant to PEGV therapy, and in patients with headaches not responsive to first-generation SRL therapy (33), we also recommend pasireotide LAR monotherapy. In special cases only, one could consider providing pasireotide LAR monotherapy in young patients with tumor concern that are not biochemically controlled with first-generation SRLs.

The PAPE study was designed to assess the efficacy and safety of pasireotide LAR (alone or in combination with PEGV) in patients who were well controlled on a combination of first-generation SRLs and PEGV. Switching to pasireotide LAR, either as monotherapy or in combination with PEGV, resulted in control of IGF-1 concentrations in most (77%) patients (34, 35). Furthermore, pasireotide LAR therapy demonstrated a PEGV sparing effect after 9 months of follow-up in 52% compared with the combination therapy with first-generation SRLs, and consequently leading to a reduction in injection frequency for patients (35). In 33 of the 45 patients from the PAPE study, tumor volume decreased but this decrease was clinically significant (≥25%) in 15 cases only (33%) (36). In both treatment groups significant improvements in quality of life were observed after switching to pasireotide LAR (37). However, we should acknowledge that assessing a difference in quality of life between monotherapy and combination therapy was not a primary endpoint of this study, and it was underpowered to detect a significant difference. In contrast to the current consensus statements that do not address the role of pasireotide in combination with PEGV (4), we propose to use this combined therapy as third-line treatment in patients without diabetes on low PEGV dosages (≤80 mg/wk) and in patients with tumor growth or symptoms of active acromegaly during first-generation SRL and PEGV combination therapy (33). Because of the PEGV sparing effect of pasireotide LAR in the former group, the PEGV dosages can be reduced or sometimes even discontinued.

Pasireotide LAR therapy is generally well tolerated and shares superimposable side effects with the first-generation SRLs, except for a greater frequency and degree of pasireotide-induced hyperglycemia and diabetes (29, 31, 34). Because the wide expression of SST_5_ receptor by pancreatic ß cells is well known, the detrimental effect on glucose homeostasis seems related to the inhibition of both insulin and incretin secretion, with a modest effect on glucagon secretion. Unfortunately, co-treatment with PEGV does not seem to offset or prevent pasireotide-induced hyperglycemia (34). It is recommended that patients considered for treatment with pasireotide LAR should be carefully screened and proactively monitored for glycemic adverse events. This early-onset proactive management should include assessing baseline fasting plasma glucose and glycosylated hemoglobin (HbA1c) levels before initiating pasireotide LAR therapy (38-41). After initiating pasireotide LAR, proactive glucose monitoring is especially important in the first 3 months of treatment (33, 39, 40). Patients without pretreatment insulin therapy had a slow onset of hyperglycemia (after the third injection of pasireotide LAR) that could be managed with oral antidiabetic medication only. On the other hand, the patients who eventually required insulin treatment were those that developed rapid hyperglycemia after the first injection of pasireotide LAR. Hence, pasireotide LAR is not recommended in patients with uncontrolled diabetes because of the high risk of developing hyperglycemia. The decision to continue pasireotide LAR treatment in those who develop hyperglycemia should be individualized. If the potential advantages of continuing pasireotide LAR outweigh the potential disadvantages of such treatment, data from healthy volunteers indicate that treatment with concomitant glucagon-like peptide 1 receptor agonists or dipeptidyl peptidase-4 inhibitors may be useful in minimizing the hyperglycemic effects (41), but data on long-term treatment with pasireotide of acromegaly are still lacking.

### Radiotherapy

Radiotherapy in patients with acromegaly is generally reserved as a third-line treatment option and is considered for those patients with biochemically persistent disease or tumor growth despite surgery or medical therapy (14, 42). Irradiation therapy, using conventional external-beam, proton-beam techniques, or stereotactic fractionated radiosurgery achieves high rates of local tumor control and moderate biochemical control in patients with acromegaly. In a single-center retrospective study of 102 patients treated with single-fraction stereotactic radiosurgery between 1990 and 2017 and followed for a median of 63 months, biochemical remission at a median of 19 months was achieved in 58 patients (57%), whereas 22 patients (22%) persisted with active disease despite additional medical therapy (43). Analysis of 352 patients from the German Acromegaly Registry, which analyzed outcomes from both stereotactic radiosurgery and fractionated radiotherapy followed for up to 45 years showed similar rates (44). The mean time to achieve normal or low IGF-1 concentrations was 2.1 (95% CI, 1.6-2.6) years for stereotactic radiosurgery and 3.0 (95% CI, 2.5-3.5) years for fractionated radiotherapy, and the 10-year remission rate was 52% and 48% for stereotactic radiosurgery and fractionated radiotherapy, respectively (44). A large study by Jenkins et al, investigating the effects of fractionated radiotherapy, demonstrated a 50% reduction of GH concentrations in about 50% of acromegaly patients in the first 2 years, a reduction up to 75% of patients after 5 years, and a further reduction up to 90% after 20 years (45). These delayed dynamics are in line with the findings of Biermasz et al, who described normalization of IGF-1 concentrations in 60% of patients at 5 years, 74% at 10 years, and even 84% after 15 years of follow-up (46). After fractionated radiotherapy of GH-producing adenomas, tumor growth arrest evaluated at 5, 10, and 15 years after treatment was 98%, 95%, and 93%, respectively (47). Reportedly, > 50% of patients with GH-producing adenomas develop hypopituitarism after fractionated radiotherapy in the long-term, which contributes to higher mortality rates (48). These patients require careful ongoing monitoring for the development of hormonal deficits. It is still unknown whether stereotactic radiosurgery is associated with a reduced impact on mortality compared with fractionated radiotherapy. Other rare side effects are visual impairment, neurocognitive deficits, and irradiation-induced secondary malignancies. Irradiation of the pituitary gland has been associated with a slight increase in mortality, possibly from cerebrovascular damage (49). The reported outcomes of patients receiving radiotherapy were only a subset of those treated for acromegaly and were often those less responsive to (repeated) surgery and medical therapies. Stereotactic fractionated radiation techniques allow for more precise treatment application with better sparing of surrounding healthy tissue and potentially resulting in lower rates of side effects. Hence, stereotactic fractionated radiotherapy should be considered the standard for pituitary radiotherapy in acromegaly.

### Controversies and Areas of Uncertainty

The molecular basis of first-generation SRL treatment resistance is poorly understood. Molecular factors leading to reduced first-generation SRL response have been proposed, such as a defective expression or genetic alterations of SST receptors and impaired signal transduction (50, 51). A correlation has been demonstrated among low SST_2_ mRNA, protein expression, and the GH-lowering response to octreotide (52, 53). The expression of SST_5_ receptor is inversely associated with the response to SRLs, and a low SST_2_/SST_5_ receptor ratio with a poor response to first-generation SRLs (54). However, marked case-to-case variations among individual tumors have been found, and some tumors are resistant to therapy despite high expression of SST_2_ receptor (55, 56). Currently, the use of these markers in histopathology is not well validated in clinical practice and is also not widely available because of cost restrictions (57). In addition, a gold standard immunohistochemical method for SST receptor detection is lacking, and the current available methods (58, 59) have been found to show high interlaboratory and interobserver agreement for SST receptor expression in neuroendocrine tumors (60). Nevertheless, if available, such factors may help to inform clinicians when making decisions about costly therapies.

There is a clear need to define the precise role and duration of presurgical medical treatment with first-generation SRLs in acromegaly patients. First-generation SRLs achieve biochemical remission in approximately 45%, whereas tumor size shrinkage is observed in up to 60% of the cases (8). Although these findings make first-generation SRLs a suitable preoperative drug for decreasing perioperative morbidity and tumor volume, as well as improving surgical outcome, current data are insufficient to support the general use of an SRL before surgery (61).

Although cabergoline is less expensive than first-generation SRLs, PEGV, and pasireotide, emerging evidence indicates that it can precipitate compulsive disorders (eg, pathological gambling, compulsive shopping, hypersexuality, binge eating), mainly through hyperactivation of the mesolimbic dopamine pathway (62, 63). This has been also confirmed by a recent study by Ozkaya et al, who state that patients with acromegaly receiving dopamine agonist treatment are at risk of developing impulse control disorders (63).

## Back to the Case

In a patient with acromegaly inadequately controlled after surgery and first-line medical therapy, adjuvant treatment needs to be tailored to achieve biochemical control, improvement of clinical symptoms, reversal of comorbidities and mortality risk, and control of local tumor effects. Given the presence of residual tumor and disease at the time of referral to our pituitary center, a decision was made to start PEGV therapy while lanreotide ATG was continued at 120.0 mg/every 4 weeks. Pasireotide LAR was not available yet for clinical use; however, pasireotide could have been considered then in this patient with tumor growth during first-generation SRL. PEGV cotreatment was initiated at a dose of 60.0 mg/wk. However, eventually a dose titration up to 700.0 mg/wk was necessary to achieve biochemical control, exceeding the highest allowed dose of 30 mg daily that is mentioned on the label. This is demonstrating that if you have access to high-dose PEGV, even patients with aggressive disease can achieve biochemical control. Of note, in this young patient with aggressive disease, pasireotide LAR would nowadays be preferred over high-dose PEGV treatment. However, a high dose of PEGV may be used in countries where pasireotide LAR is not available, or when a brittle diabetes mellitus is present. The role of radiotherapy was discussed with this patient; however, because of the patient’s personal preference he did not receive this treatment. Nine years later, pasireotide LAR became available for treatment of patients with acromegaly and he participated in a study to investigate pasireotide LAR alone and in combination with PEGV (PAPE study (34, 35)). We decided to start pasireotide LAR in combination with PEGV to determine whether it could reduce PEGV dose and preserve biochemical and tumor control. Pasireotide LAR was initiated at a dose of 60.0 mg every 4 weeks, whereas PEGV was continued but according to study protocol reduced by 50% to 350.0 mg/wk. Eventually up-titration of PEGV after 3 months to 540.0 mg/wk was necessary to achieve IGF-1 normalization. HbA1c levels increased from 36 mmol/mol (normal value, <42 mmol/mol) before the start of pasireotide LAR to 44 mmol/mol after 6 months of treatment and he was placed on antidiabetic therapy with metformin 3000 mg/d (ie, highest allowed dose), gliclazide 60 mg/d, and liraglutide 1.8 mg/d. Pasireotide LAR in combination with PEGV markedly reduced the tumor size: the tumor volume reduced from 8086 mm^3^ before initiation of pasireotide to 5896 mm^3^ during 15 months of treatment (Fig. 1C, 1D). Interestingly, the quantification of the T2-weighted MRI signal by region of interest measurement showed in > 50% of the adenoma a higher T2 signal intensity ratio during treatment: region of interest adenoma/gray matter ratio was 1.2 at baseline and increased to 2.0 during 15 months of pasireotide LAR treatment in combination with PEGV (Fig. 1C, 1D). In general, heterogeneity on T1- and a T2-hyperintense signal indicates cystic degeneration, tumor cell necrosis, or both, which suggest an antitumor effect of pasireotide LAR. SST receptor subtyping in the tumor specimen was evaluated using an IRS (59, 64), showing membranous expression of IRS 1 for SST_2a_ receptor, IRS 12 for SST_5_ receptor, and SST_2_/SST_5_ receptor ratio of 0.08. SST_3_ receptor was not evaluated. Although SST_3_ receptor might be responsible for the pasireotide LAR-induced cystic degeneration, tumor cell necrosis, or both, currently antibody assessment for SST_3_ is not that robust as for SST_2_ and SST_5_ receptor, and SST_3_ staining can be cumbersome. Studying the effects of pasireotide-LAR on pituitary histology might help unravel the differences in SST receptors specific signaling pathways and should be further explored. Recently, because of further worsening of glycemic control (HbA1c 82 mmol/mol, normal value < 42 mmol/mol), pasireotide was discontinued after 53 months of pasireotide LAR treatment in combination with PEGV. At the last follow-up, the patient was treated with lanreotide ATG 120.0 mg every 4 weeks in combination with PEGV 240.0 mg/wk; IGF-1 levels and tumor size remained controlled.

## Data Availability

Restrictions apply to the availability of some or all data generated or analyzed during this study to preserve patient confidentiality. The corresponding author will on request detail the restrictions and any conditions under which access to some data may be provided.

## Abbreviations

ATG: autogel
HbA1c: glycated hemoglobin
IRS: immunoreactivity score
LAR: long-acting release
MRI: magnetic resonance imaging
PEGV: pegvisomant
SRL: somatostatin receptor ligand
SST: somatostatin
ULN: upper limit of normal

## References

[1] Petrossians P, Borges-Martins L, Espinoza C, et al Gross total resection or debulking of pituitary adenomas improves hormonal control of acromegaly by somatostatin analogs. Eur J Endocrinol. 2005;152(1):61-66. 15762188 10.1530/eje.1.01824

[2] Melmed S . Acromegaly pathogenesis and treatment. J Clin Invest. 2009;119(11):3189-3202. 19884662 10.1172/JCI39375 PMC2769196

[3] Giustina A, Barkan A, Beckers A, et al A consensus on the diagnosis and treatment of acromegaly comorbidities: an update. J Clin Endocrinol Metab. 2020;105(4):e937-ee46. 10.1210/clinem/dgz096 31606735

[4] Melmed S, Bronstein MD, Chanson P, et al A Consensus Statement on acromegaly therapeutic outcomes. Nat Rev Endocrinol. 2018;14(9):552-561. 30050156 10.1038/s41574-018-0058-5 PMC7136157

[5] Katznelson L, Laws ER Jr, Melmed S, et al Acromegaly: an Endocrine Society clinical practice guideline. J Clin Endocrinol Metab. 2014;99(11):3933-3951. 25356808 10.1210/jc.2014-2700

[6] Coopmans EC, Postma MR, Wolters TLC, et al Predictors for remission after transsphenoidal surgery in acromegaly: a Dutch multicenter study. J Clin Endocrinol Metab. 2021;106(6):1783-1792. 33544833 10.1210/clinem/dgab069 PMC8118364

[7] Babu H, Ortega A, Nuno M, et al Long-term endocrine outcomes following endoscopic endonasal transsphenoidal surgery for acromegaly and associated prognostic factors. Neurosurgery. 2017;81(2):357-366. 28368500 10.1093/neuros/nyx020

[8] Caron PJ, Bevan JS, Petersenn S, et al Tumor shrinkage with lanreotide Autogel 120 mg as primary therapy in acromegaly: results of a prospective multicenter clinical trial. J Clin Endocrinol Metab. 2014;99(4):1282-1290. 24423301 10.1210/jc.2013-3318 PMC4009579

[9] Petrossians P, Tichomirowa MA, Stevenaert A, Martin D, Daly AF, Beckers A. The Liege Acromegaly Survey (LAS): a new software tool for the study of acromegaly. Ann Endocrinol (Paris). 2012;73(3):190-201. 22682917 10.1016/j.ando.2012.05.001

[10] Buchfelder M . Treatment of pituitary tumors: surgery. Endocrine. 2005;28(1):67-75. 16311412 10.1385/ENDO:28:1:067

[11] Buchfelder M, Schlaffer SM. Novel techniques in the surgical treatment of acromegaly: applications and efficacy. Neuroendocrinology. 2016;103(1):32-41. 26536097 10.1159/000441980

[12] Mathioudakis N, Salvatori R. Management options for persistent postoperative acromegaly. Neurosurg Clin N Am. 2012;23(4):621-638. 23040748 10.1016/j.nec.2012.06.005 PMC6031123

[13] Sandret L, Maison P, Chanson P. Place of cabergoline in acromegaly: a meta-analysis. J Clin Endocrinol Metab. 2011;96(5): 1327-1335. 21325455 10.1210/jc.2010-2443

[14] Katznelson L, Atkinson JLD, Cook DM, Ezzat SZ, Hamrahian AH, Miller KK. American Association of Clinical Endocrinologists medical guidelines for clinical practice for the diagnosis and treatment of acromegaly-2011 update. Endocr Pract. 2011;17 (4):636-646. 10.4158/ep.17.4.636 21846619

[15] Kuhn E, Chanson P. Cabergoline in acromegaly. Pituitary. 2017;20(1):121-128. 28025719 10.1007/s11102-016-0782-6

[16] Trainer PJ, Drake WM, Katznelson L, et al Treatment of acromegaly with the growth hormone-receptor antagonist pegvisomant. N Engl J Med. 2000;342(16):1171-1177. 10770982 10.1056/NEJM200004203421604

[17] van der Lely A, J, Hutson RK, Trainer PJ, et al Long-term treatment of acromegaly with pegvisomant, a growth hormone receptor antagonist. Lancet. 2001;358(9295):1754-1759. 11734231 10.1016/s0140-6736(01)06844-1

[18] van der Lely A, J, Jonsson P, et al Treatment with high doses of pegvisomant in 56 patients with acromegaly: experience from ACROSTUDY. Eur J Endocrinol. 2016;175(4):239-245. 27401863 10.1530/EJE-16-0008

[19] Barkan AL, Burman P, Clemmons DR, et al Glucose homeostasis and safety in patients with acromegaly converted from long-acting octreotide to pegvisomant. J Clin Endocrinol Metab. 2005;90(10):5684-5691. 16076947 10.1210/jc.2005-0331

[20] Feola T, Cozzolino A, Simonelli I, et al Pegvisomant improves glucose metabolism in acromegaly: a meta-analysis of prospective interventional studies. J Clin Endocrinol Metab. 2019;104(7):2892-2902. 30869797 10.1210/jc.2018-02281

[21] Buchfelder M, van der Lely A, J, Biller BMK, et al Long-term treatment with pegvisomant: observations from 2090 acromegaly patients in ACROSTUDY. Eur J Endocrinol. 2018;179(6):419-427. 30325178 10.1530/EJE-18-0616

[22] Neggers SJ, Franck SE, de Rooij F, W, et al Long-term efficacy and safety of pegvisomant in combination with long-acting somatostatin analogs in acromegaly. J Clin Endocrinol Metab. 2014;99(10):3644-3652. 24937542 10.1210/jc.2014-2032

[23] Fleseriu M, Führer-Sakel D, van der Lely AJ, et al More than a decade of real-world experience of pegvisomant for acromegaly: ACROSTUDY. Eur J Endocrinol. 2021;185(4):525-538. 34342594 10.1530/EJE-21-0239 PMC8428076

[24] Neggers SJ, van Aken MO, Janssen JA, et al Long-term efficacy and safety of combined treatment of somatostatin analogs and pegvisomant in acromegaly. J Clin Endocrinol Metab. 2007;92(12):4598-4601. 17895318 10.1210/jc.2007-1234

[25] Trainer PJ, Ezzat S, D’Souza GA, Layton G, Strasburger CJ. A randomized, controlled, multicentre trial comparing pegvisomant alone with combination therapy of pegvisomant and long-acting octreotide in patients with acromegaly. Clin Endocrinol (Oxf). 2009;71(4):549-557. 19438906 10.1111/j.1365-2265.2009.03620.x

[26] Neggers SJ, van der Lely A, J. Combination treatment with somatostatin analogues and pegvisomant in acromegaly. Growth Horm IGF Res. 2011;21(3):129-133. 21498099 10.1016/j.ghir.2011.03.004

[27] Bonert V, Mirocha J, Carmichael J, Yuen KCJ, Araki T, Melmed S. Cost-effectiveness and efficacy of a novel combination regimen in acromegaly: a prospective, randomized trial. J Clin Endocrinol Metab. 2020;105(9):e3236-e3e45. 10.1210/clinem/dgaa444 32754748

[28] Neggers SJCMM, van Aken MO, de Herder W, W, et al Quality of life in acromegalic patients during long-term somatostatin analog treatment with and without pegvisomant. J Clin Endocrinol Metab. 2008;93(10):3853-3859. 18647806 10.1210/jc.2008-0669

[29] Colao A, Bronstein MD, Freda P, et al Pasireotide versus octreotide in acromegaly: a head-to-head superiority study. J Clin Endocrinol Metab. 2014;99(3):791-799. 24423324 10.1210/jc.2013-2480 PMC3965714

[30] Gadelha M, M B, Colao A, et al Evaluation of the efficacy and safety of switching to pasireotide in patients with acromegaly inadequately controlled with first-generation somatostatin analogs. Front Endocrinol (Lausanne). 2019;10(931):931. 32117045 10.3389/fendo.2019.00931 PMC7008501

[31] Gadelha MR, Bronstein MD, Brue T, et al Pasireotide versus continued treatment with octreotide or lanreotide in patients with inadequately controlled acromegaly (PAOLA): a randomised, phase 3 trial. Lancet Diabetes Endocrinol. 2014;2(11):875-884. 25260838 10.1016/S2213-8587(14)70169-X

[32] Bronstein MD, Fleseriu M, Neggers S, et al Switching patients with acromegaly from octreotide to pasireotide improves biochemical control: crossover extension to a randomized, double-blind, phase III study. BMC Endocr Disord. 2016;16:16. 27039081 10.1186/s12902-016-0096-8 PMC4818908

[33] Coopmans EC, Muhammad A, van der Lely A, J, Janssen J, Neggers S. How to position pasireotide LAR treatment in acromegaly. J Clin Endocrinol Metab. 2019;104(6):1978-1988. 30608534 10.1210/jc.2018-01979

[34] Muhammad A, van der Lely A, J, Delhanty PJD, et al Efficacy and safety of switching to pasireotide in patients with acromegaly controlled with pegvisomant and first-generation somatostatin analogues (PAPE Study). J Clin Endocrinol Metab. 2018;103(2):586-595. 29155991 10.1210/jc.2017-02017

[35] Muhammad A, Coopmans EC, Delhanty PJD, et al Efficacy and safety of switching to pasireotide in acromegaly patients controlled with pegvisomant and somatostatin analogues: PAPE extension study. Eur J Endocrinol. 2018;179(5):269-277. 30076159 10.1530/EJE-18-0353

[36] Coopmans EC, Schneiders JJ, El-Sayed N, et al T2-signal intensity, SSTR expression, and somatostatin analogs efficacy predict response to pasireotide in acromegaly. Eur J Endocrinol. 2020;182(6):595-605. 32375119 10.1530/EJE-19-0840

[37] Coopmans EC, El-Sayed N, Frystyk J, et al Soluble Klotho: a possible predictor of quality of life in acromegaly patients. Endocrine. 2020;69(1):165-174. 32333268 10.1007/s12020-020-02306-4 PMC7343750

[38] Luger A . Hyperglycemia in pasireotide-treated patients with acromegaly and its treatment. Endocrine. 2016;54(1):1-2. 27388591 10.1007/s12020-016-1029-z

[39] Samson SL . Management of hyperglycemia in patients with acromegaly treated with pasireotide LAR. Drugs. 2016;76(13):1235-1243. 27473537 10.1007/s40265-016-0615-y

[40] Wildemberg LE, Gadelha MR. Pasireotide for the treatment of acromegaly. Expert Opin Pharmacother. 2016;17(4):579-588. 26808354 10.1517/14656566.2016.1146688

[41] Breitschaft A, Hu K, Hermosillo Resendiz K, Darstein C, Golor G. Management of hyperglycemia associated with pasireotide (SOM230): healthy volunteer study. Diabetes Res Clin Pract. 2014;103(3):458-465. 24461109 10.1016/j.diabres.2013.12.011

[42] Hannon MJ, Barkan AL, Drake WM. The role of radiotherapy in acromegaly. Neuroendocrinology. 2016;103(1):42-49. 26088716 10.1159/000435776

[43] Graffeo CS, Donegan D, Erickson D, et al The impact of insulin-like growth factor index and biologically effective dose on outcomes after stereotactic radiosurgery for acromegaly: cohort study. Neurosurgery. 2020;87(3):538-546. 32267504 10.1093/neuros/nyaa054 PMC7426191

[44] Knappe UJ, Petroff D, Quinkler M, et al Fractionated radiotherapy and radiosurgery in acromegaly: analysis of 352 patients from the German Acromegaly Registry. Eur J Endocrinol. 2020;182(3):275-284. 31917680 10.1530/EJE-19-0784

[45] Jenkins PJ, Bates P, Carson MN, Stewart PM, Wass JA. Conventional pituitary irradiation is effective in lowering serum growth hormone and insulin-like growth factor-I in patients with acromegaly. J Clin Endocrinol Metab. 2006;91(4):1239-1245. 16403824 10.1210/jc.2005-1616

[46] Biermasz NR, van Dulken H, Roelfsema F. Long-term follow-up results of postoperative radiotherapy in 36 patients with acromegaly. J Clin Endocrinol Metab. 2000;85(7):2476-2482. 10902796 10.1210/jcem.85.7.6699

[47] Minniti G, Jaffrain-Rea ML, Osti M, et al The long-term efficacy of conventional radiotherapy in patients with GH-secreting pituitary adenomas. *Clin Endocrinol (Oxf).* 2005; 62(2): 210-216. 10.1111/j.1365-2265.2005.02199.x 15670198

[48] Sherlock M, Reulen RC, Alonso AA, et al ACTH deficiency, higher doses of hydrocortisone replacement, and radiotherapy are independent predictors of mortality in patients with acromegaly. J Clin Endocrinol Metab. 2009;94(11):4216-4223. 19808848 10.1210/jc.2009-1097

[49] Sherlock M, Ayuk J, Tomlinson JW, et al Mortality in patients with pituitary disease. Endocr Rev. 2010;31(3):301-342. 20086217 10.1210/er.2009-0033

[50] Ibanez-Costa A, Korbonits M. AIP and the somatostatin system in pituitary tumours. J Endocrinol. 2017;235(3):R101-RR16. 28835453 10.1530/JOE-17-0254

[51] Larkin S, Reddy R, Karavitaki N, Cudlip S, Wass J, Ansorge O. Granulation pattern, but not GSP or GHR mutation, is associated with clinical characteristics in somatostatin-naive patients with somatotroph adenomas. Eur J Endocrinol. 2013;168(4):491-499. 23288882 10.1530/EJE-12-0864

[52] Taboada GF, Neto LV, Luque RM, et al Impact of gsp oncogene on the mRNA content for somatostatin and dopamine receptors in human somatotropinomas. Neuroendocrinology. 2011;93(1):40-47. 21079388 10.1159/000322040

[53] Chalabi M, Duluc C, Caron P, et al Somatostatin analogs: does pharmacology impact antitumor efficacy? Trends Endocrinol. Metab. 2014;25(3):115-127. 24405892 10.1016/j.tem.2013.11.003

[54] Gatto F, Feelders RA, Franck SE, et al In vitro head-to-head comparison between octreotide and pasireotide in GH-secreting pituitary adenomas. J Clin Endocrinol Metab. 2017;102(6):2009-2018. 28323931 10.1210/jc.2017-00135

[55] Ferone D, de Herder W, W, Pivonello R, et al Correlation of in vitro and in vivo somatotropic adenoma responsiveness to somatostatin analogs and dopamine agonists with immunohistochemical evaluation of somatostatin and dopamine receptors and electron microscopy. J Clin Endocrinol Metab. 2008;93(4):1412-1417. 18211974 10.1210/jc.2007-1358

[56] Colao A, Auriemma RS, Lombardi G, Pivonello R. Resistance to somatostatin analogs in acromegaly. Endocr Rev. 2011;32(2):247-271. 21123741 10.1210/er.2010-0002

[57] Gadelha MR, Kasuki L, Korbonits M. Novel pathway for somatostatin analogs in patients with acromegaly. Trends Endocrinol Metab. 2013;24(5):238-246. 23270713 10.1016/j.tem.2012.11.007

[58] Volante M, Brizzi MP, Faggiano A, et al Somatostatin receptor type 2A immunohistochemistry in neuroendocrine tumors: a proposal of scoring system correlated with somatostatin receptor scintigraphy. Mod Pathol. 2007;20(11):1172-1182. 17873898 10.1038/modpathol.3800954

[59] Remmele W, Stegner HE. Recommendation for uniform definition of an immunoreactive score (IRS) for immunohistochemical estrogen receptor detection (ER-ICA) in breast cancer tissue. Pathologe. 1987;8(3):138-140. 3303008

[60] Kasajima A, Papotti M, W I, et al High interlaboratory and interobserver agreement of somatostatin receptor immunohistochemical determination and correlation with response to somatostatin analogs. Hum Pathol. 2018;72: 144-152. 29180250 10.1016/j.humpath.2017.11.008

[61] Fleseriu M, Hoffman AR, Katznelson L, AACE Neuroendocrine and Pituitary Scientific Committee. American Association of Clinical Endocrinologists and American College of Endocrinology Disease State Clinical Review: management of acromegaly patients: what is the role of pre-operative medical therapy? Endocr Pract. 2015;21(6):668-673. 26135961 10.4158/EP14575.DSCR

[62] Abler B, Hahlbrock R, Unrath A, Grön G, Kassubek J. At-risk for pathological gambling: imaging neural reward processing under chronic dopamine agonists. Brain. 2009;132(Pt 9):2396-2402. 19567700 10.1093/brain/awp170

[63] Ozkaya HM, Sahin S, Korkmaz OP, et al Patients with acromegaly might not be at higher risk for dopamine agonist-induced impulse control disorders than those with prolactinomas. Growth Horm IGF Res. 2020;55:101356. 33010581 10.1016/j.ghir.2020.101356

[64] Gatto F, Feelders RA, van der Pas R, et al Immunoreactivity score using an anti-sst2A receptor monoclonal antibody strongly predicts the biochemical response to adjuvant treatment with somatostatin analogs in acromegaly. J Clin Endocrinol Metab. 2013;98(1):E66-E71. 23118420 10.1210/jc.2012-2609

